# Effect of Acupoint Stimulation on Controlling Pain from Heel Lance in Neonates: A Systematic Review and Meta-Analysis of Randomized Controlled Trials

**DOI:** 10.3390/children10061024

**Published:** 2023-06-07

**Authors:** Sio-Ian Tou, Chia-Yu Huang, Hung-Rong Yen

**Affiliations:** 1 Department of Pediatrics, Chung Kang Branch, Cheng-Ching General Hospital, Taichung 407, Taiwan; 2 Department of Family Medicine, Taichung Tzu Chi Hospital, Buddhist Tzu Chi Medical Foundation, Taichung 427, Taiwan; tc1811201@tzuchi.com.tw; 3 Graduate Institute of Chinese Medicine, School of Chinese Medicine, College of Chinese Medicine, China Medical University, Taichung 404, Taiwan; 4 International Master Program in Acupuncture, College of Chinese Medicine, China Medical University, Taichung 404, Taiwan; 5 Department of Chinese Medicine, China Medical University Hospital, Taichung 404, Taiwan; 6 School of Post-Baccalaureate Chinese Medicine, College of Chinese Medicine, China Medical University, Taichung 404, Taiwan; 7 Research Center for Traditional Chinese Medicine, Department of Medical Research, China Medical University Hospital, Taichung 404, Taiwan; 8 Chinese Medicine Research Center, China Medical University, Taichung 404, Taiwan

**Keywords:** acupoint stimulation, pain, heel lance, neonates, meta-analysis

## Abstract

To evaluate the effect of acupoint stimulation compared to other interventions on pain control in neonates who underwent heel lance, we searched for randomized controlled trials across six databases (CINAHL, Cochrane Library, EMBASE, Medline, PubMed, and Web of Science) published up to January 2023. Studies comparing acupoint stimulation and other interventions for controlling heel lance pain in neonates were included. These reports measured at least one of the following variables: pain score, crying time, oxygenation saturation, heart rate, respiration rate, and duration of the procedure. The data were independently extracted by two authors, and the PRISMA guidelines for study selection were followed. A total of 79 articles were screened, and 10 studies, with results on 813 neonates, were included in the final selection. The pain scores recorded during the heel lance procedure were not significantly different between the acupoint stimulation cohort and the control cohort (SMD of −0.26, 95% confidence interval (CI) from −0.52 to 0.01; *p* = 0.06; I^2^ = 68%). After processing the subgroup analyses, significant differences were found in the comparisons of acupuncture vs. usual care (SMD of −1.25, 95% CI from −2.23 to 0.27) and acupressure vs. usual care (SMD of −0.62, 95% CI from −0.96 to −0.28); nonsignificant differences were found in other comparisons. Our results demonstrate that acupoint stimulation may improve pain score during the heel lance procedure.

## 1. Introduction

The heel lance procedure is commonly used to collect blood from neonates for sampling [1]. A sterile lancet is used to penetrate the skin on the lateral side of the foot, which can cause the neonate discomfort in the form of pain [2], manifesting as crying, irritability, increased heart rate, increased respiratory rate, and the need for oxygenation [3]. Although the pain score of venipuncture is lower than that of the heel lance in some regions, the heel lance is still the standard procedure for neonates [4]. Furthermore, in neonatal intensive care units, some patients may be subject to heel lancing several times for close monitoring [2]. Researchers have noted the long-term negative effect of this painful procedure on neurological development in neonates [5,6]. Thus, preventive measures, such as sweet-tasting solutions [7], non-nutritive sucking [8], position change [9], kangaroo care [10], and music [11], have been studied in an attempt to reduce the pain experienced during this and other procedures performed on neonates. Most of these interventions are non-pharmaceutical approaches, and the therapeutic differences between them are still under study [4]. One of the integrative interventions, acupoint stimulation (APS), has been studied in clinical trials for several decades to investigate its analgesic effect in adults [12]. APS is mentioned less in studies that recruit children [13]. 

APS has been used for thousands of years to control symptoms and treat disease [14,15]. APS is practiced, according to the theory of traditional Chinese medicine (TCM), through acupuncture (sterile needle stimulation) [14], acupressure (manual stimulation) [16], laser acupuncture [17], electrical stimulation [18], and magnetic acupuncture (stimulation with specific equipment) and involves stimulating the acupoints of the human body to achieve therapeutic effects, such as pain control. The World Health Organization suggests that procedural pain could be treated by APS [19]. In the adult population, evaluations of the effects of APS to relieve pain through meta-analyses have been conducted [12]. Previous randomized control trials (RCTs) have also shown that APS could improve pain from a tonsillectomy, venipuncture, headache, tooth pain, and appendectomy in children [16,17,18,19,20]. Considering that it is a noninvasive therapy, APS may be more suitable for use in neonates. RCTs have used APS interventions to decrease the pain induced by heel lance procedures in neonates [21].

However, most reviews and meta-analyses have not reported the effect of pain control by APS in children [13], and evidence regarding other non-pharmaceutical interventions’ ability to relieve pain is accumulating. Thus, an evidence-based evaluation of the effect of APS on children is needed, especially for those who may undergo routine procedures such as a heel lance. The aim of this study was to reveal the ability of APS to improve pain from a heel lance in neonates through meta-analysis. Our results will be useful for caregivers and medical staff interested in APS and will help researchers design their studies more comprehensively to reveal the role of APS in controlling pain in neonates in the future.

## 2. Materials and Methods

### 2.1. Search Strategy

The registration for this study was published in PROSPERO (no. CRD42023398898). Six databases (CINAHL, Cochrane Library, EMBASE, Medline, PubMed, and Web of Science) were searched for RCTs published up to January 2023 [1,8]. The search terms used were as follows: (neonate OR newborn OR infant OR baby OR Premature OR Preterm OR Term OR Prematurity) AND (((heel lancing) OR (heel lance)) OR (heel stick)) OR (heel prick) AND ((((acupuncture) OR (acupressure)) OR (electroacupuncture)) OR (ear acupoint)) OR (auricular point). The papers that cited potentially eligible studies were searched. An additional search of the listed references of the included studies was carried out. Two independent authors (S.-I.T., C.-Y.H.) assessed the eligibility of the studies by reading the full text, and disagreements were resolved by discussion with the other author (H.-R.Y.). 

### 2.2. Selection Criteria

The study selection was performed following the Preferred Reporting Items for Systematic Reviews and Meta-Analyses (PRISMA) guidelines (Figure S1). A study was included if (1) the study design was an RCT; (2) the intervention arms included APS; (3) the study population was neonates; and (4) the outcome reports included ≥1 item from the primary, secondary, or tertiary outcomes listed below. A study was excluded if (1) the study design was non-RCT; (2) the population was nonhuman or not neonates; (3) APS was not included in the intervention arm; or (4) the results did not report any of the outcomes listed below.

The primary outcomes included were pain scores, including the Premature Infant Pain Profile (PIPP) [21,22,23,24,25], the Neonatal Infant Pain Scale (NIPS) [26,27,28,29], and the Neonatal Pain, Agitation, and Sedation Scale (N-PASS) [30], which are commonly used tools to evaluate the degree of pain in neonates. Secondary outcomes included were regarding the presence of established evidence of physical responses to the painful procedure, including crying time, oxygenation saturation, heart rate, and respiration rate [26]. The tertiary outcome of interest was the duration of the procedure [27]. We used Pearson/Spearman correlation (r), intraclass correlation coefficient (ICC), or Cronbach’s alpha coefficient (α) to present the reliability and validity of the tools of pain evaluation and behavior indicators. ICC ≥ 0.70, r ≥ 0.80, and α ≥ 0.80 indicate high reliability. α values of 0.70, 0.60, and 0.50 are considered to indicate acceptable, marginal, and poor reliability, respectively. ICC ≥ 0.50 and r ≥ 0.70 indicate high validity.

### 2.3. Data Extraction

The data extraction was independently performed by two authors (S.-I.T. and validated by C.-Y.H.), and disagreements were resolved by discussion between all authors. The studies’ authors; region in which the trial was practiced; publication year; maturity of neonates; prescriptions of APS and control cohorts; evaluation tools for outcomes; outcomes for pain measures, including pain scores, crying time, oxygenation concentration, heart rate, and respiratory rate; and duration of the heel lance were recorded.

### 2.4. Risk of Bias Assessment

Two independent authors (S.-I.T. and H.-R.Y.) followed the suggestions of the Cochrane Handbook to assess the risk of bias in the included studies, and disagreements were resolved by consensus between all authors [31]. There are 6 items in the Cochrane risk of bias tool (selection bias, performance bias, detection bias, attrition bias, reporting bias, and other bias), and the reviewers rated each included study as “low risk of bias”, “unclear risk of bias”, or “high risk of bias”. A graph and a table were generated to present the risk of bias.

### 2.5. Statistical Analysis

We used the software Review Manager 5.3.0 to perform our meta-analyses, STATA 17.0 to process meta-regression, and STATA to perform the meta-analyses and meta-regression. The homogenous outcomes of the included studies were pooled by Review Manager. Table 1 presents the characteristics of the included studies. When the data belonged to a continuous variable, the mean difference and standard deviation (SD) were calculated and presented [32]. When the evaluation tool was the same, the weighted mean difference (WMD) was used to measure the difference between two cohorts, or the standardized mean difference (SMD) was determined. The heterogeneity of each pooling was examined by the I^2^ test: I^2^ ≥ 75%, 50–75%, and ≤50% were considered high, moderate, and low levels of heterogeneity, respectively [32]. If I^2^ ≥ 75%, the random effects model was used. The treatment differences are illustrated by forest plots.

The funnel plots were generated by Review Manager to present the publication bias. If asymmetry was noted in a funnel plot, publication bias may have existed. Egger’s test was used to confirm the existence of publication bias [33]. If *p* < 0.05 was noted from the result of Egger’s test, this indicated publication bias. The trim and fill method was used when the publication bias could not be calculated by the other methods [34].

## 3. Results

### 3.1. Characteristics of RCTs

In total, 10 RCTs (*n* = 813) met the inclusion criteria and were included in the final analysis [21,22,23,24,25,26,27,28,29,30]. There were 336 neonates in the APS cohorts and 477 neonates in the control cohorts (non-APS and usual care) (Table 1). The primary, secondary, and tertiary outcomes of the included studies are presented in Section 3.2, Section 3.3, and Section 3.4, respectively. The bias scores of the included studies are shown in Figure S2. Six studies used random sequence generation [23,24,25,27,28,30], five studies used allocation concealment [24,25,27,28,30], one study employed blinding of the participants and personnel [27], three studies employed blinding of the outcome assessment [24,25,27], ten studies were found to have a low risk of incomplete outcome data [22,23,24,25,26,27,28,29,30], and nine studies had a low risk of selective reporting [21,22,24,25,26,27,28,29,30].

### 3.2. Primary Outcomes

#### 3.2.1. Pain Score during Heel Lance

**Figure 1:**
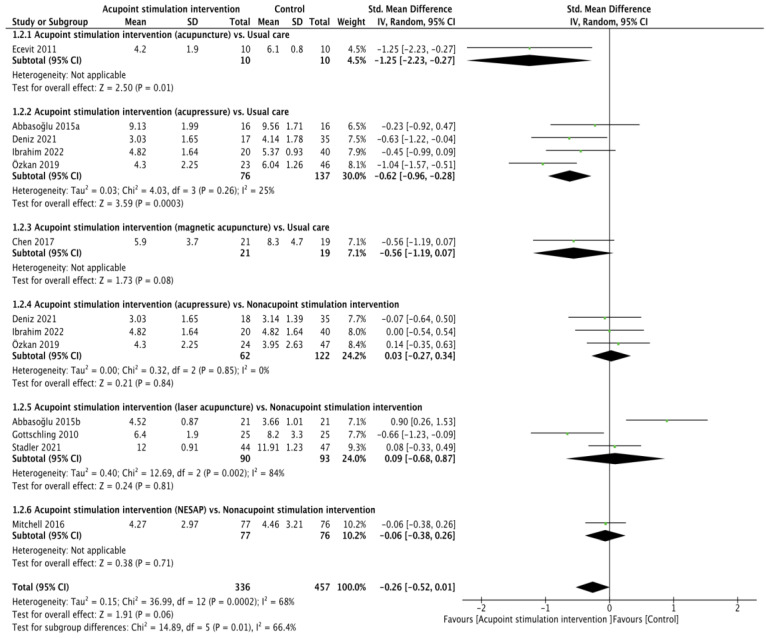
Forest plot of pain score during heel lance procedure [21,22,23,24,25,26,27,28,29,30].

All ten RCTs [21,22,23,24,25,26,27,28,29,30] presented heel lance pain scores (validity: r = 0.93 for the NIPS [35], ICC from 0.86 to 0.93 for the PIPP [36], r  = 0.874 and α = 0.84 to 0.89 for the N-PASS [37]; reliability: r = 0.868 for the NIPS [38], ICC from 0.94 to 0.98 and α = 0.71 for the PIPP [36,39], ICC from 0.86 to 0.93 for the N-PASS) [37]. The pain score between APS and the control cohort was not found to be significant through pooling with the random effects model (SMD of −0.26, 95% confidence interval (CI) from −0.52 to 0.01; *p* = 0.06; I^2^ = 68%). The results of the subgroup analyses showed that the differences were not obvious in the comparisons of acupressure vs. usual care (SMD of −0.56, 95% CI from −1.19 to 0.07; *p* = 0.08), acupressure vs. nonacupoint stimulation intervention (SMD of 0.03, 95% CI from −0.27 to 0.34; *p* = 0.84; I^2^ = 0%), laser acupuncture vs. nonacupoint stimulation intervention (SMD of 0.09, 95% CI from −0.68 to 0.87; *p* = 0.81; I^2^ = 84%), or NESAP vs. nonacupoint stimulation intervention (SMD of −0.06, 95% CI from −0.38 to 0.26; *p* = 0.71), but the differences were significant in the comparisons of acupuncture vs. usual care (SMD of −1.25, 95% CI from −2.23 to 0.27; *p* = 0.01) and acupressure vs. usual care (SMD of −0.62, 95% CI from −0.96 to −0.28; *p* = 0.0003; I^2^ = 25%, Figure 1).

#### 3.2.2. Pain Score after Heel Lance

**Figure 2:**
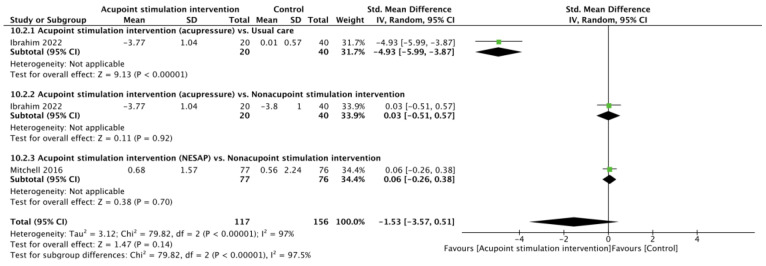
Forest plot of pain score after heel lance procedure [23,29].

Two RCTs [23,29] reported pain scores after heel lance. There was no significant difference in pain scores between the APS and control cohorts (SMD of −1.53, 95% CI from −3.57 to 0.51; *p* = 0.14; I^2^ = 97%) according to the random effects model. Subgroup analyses showed that the differences were not significant in comparisons of the acupressure vs. nonacupoint stimulation intervention (SMD of 0.03, 95% CI from −0.51 to 0.57; *p* = 0.92) or the NESAP vs. nonacupoint stimulation intervention (SMD of 0.06, 95% CI from −0.26 to 0.38; *p* = 0.70), but a significantly lower pain score was noted for acupressure when comparing it with usual care (SMD of −4.93, 95% CI from −5.99 to −3.87; *p* < 0.00001, Figure 2).

### 3.3. Secondary Outcomes

#### 3.3.1. Crying Time

**Figure 3:**
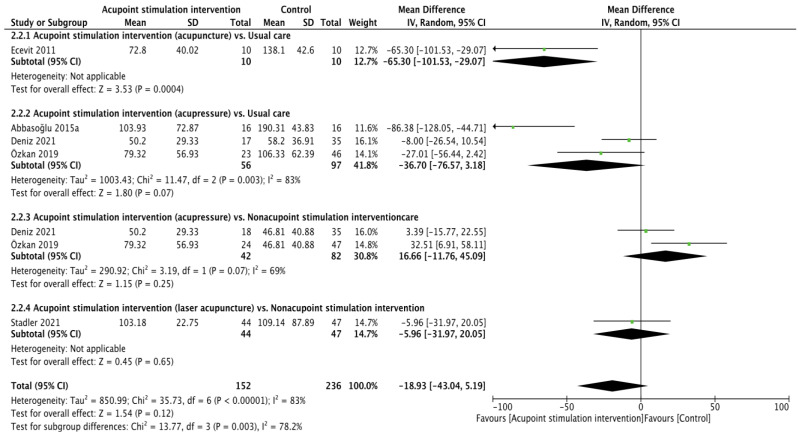
Forest plot of crying time [22,25,26,28,30].

Crying time (reliability: ICC = 0.905–0.945 [40], validity: r = 0.976 [35]) was reported in five RCTs [22,25,26,28,30], and the pooling results were not significant according to calculations made with the random effects model (WMD of −18.93 (second), 95% CI from −43.04 to 5.19; *p* = 0.12; I^2^ = 83%). Subgroup analyses showed that acupuncture resulted in less crying time in comparison to usual care (WMD of −65.30 (second), 95% CI from −101.53 to −29.07; *p* = 0.0004). The differences in crying time recorded when comparing acupressure vs. usual care (WMD of −36.70 (second), 95% CI −76.57 to 3.18; *p* = 0.07; I^2^ = 83%), acupressure vs. usual care nonacupoint stimulation intervention (WMD of 16.66 (second), 95% CI from −11.76 to 45.09; *p* = 0.25; I^2^ = 69%), and laser acupuncture vs. nonacupoint stimulation intervention (WMD of −5.96 (second), 95% CI from −31.97 to 20.05; *p* = 0.65) were not significant (Figure 3).

#### 3.3.2. Oxygenation Saturation during Heel Lance

Three RCTs [24,28,29] reported their outcomes regarding oxygenation saturation during heel lance using the fixed effects model, and the results of pooling revealed lower oxygenation desaturation in the APS cohort (WMD of 1.09 (%), 95% CI from 0.28 to 1.89; *p* = 0.008; I^2^ = 25%). Significant differences were found only in the comparison of acupressure vs. usual care (WMD of 1.95 (%), 95% CI from 0.81 to 3.10; *p* = 0.0008; I^2^ = 0%). No significant differences were found in the comparisons of magnetic acupuncture (WMD of 1.40 (%), 95% CI from −1.88 to 4.68; *p* = 0.40) vs. usual care, or acupuncture vs. nonacupoint stimulation intervention (WMD of 0.08 (%), 95% CI from −1.13 to 1.29; *p* = 0.89; I^2^ = 0%, Figure S3).

#### 3.3.3. Oxygenation Saturation after Heel Lance

Five RCTs [24,25,26,28,29] presented results regarding oxygenation saturation after heel lance, but there was no significant difference in oxygenation saturation after heel lance in the APS cohort compared with the control cohort (fixed effects model, WMD of 0.23 (%), 95% CI from −0.54 to 0.99; *p* = 0.56; I^2^ = 0%, Figure S4). In the subgroup analyses, no significant differences were found in the comparisons of acupuncture vs. usual care (WMD of 0.50 (%), 95% CI from -2.39 to 3.39; *p* = 0.73), acupressure vs. usual care (WMD of −0.11 (%), 95% CI from −1.48 to 1.26; *p* = 0.87; I^2^ = 0%), magnetic acupuncture vs. usual care (WMD of 0.00 (%), 95% CI from −3.62 to 3.62; *p* = 1.00), acupressure vs. nonacupoint stimulation intervention (WMD of 1.15 (%), 95% CI from −0.29 to 2.58; *p* = 0.12), or laser acupuncture vs. nonacupoint stimulation intervention (WMD of −0.35 (%), 95% CI from −1.78 to 1.08; *p* = 0.63). 

#### 3.3.4. Heart Rate during Heel Lance

Three RCTs [24,28,29] presented results regarding the heart rate during the heel lance, and the difference between the APS and control cohorts did not achieve statistical significance after pooling using the fixed effects model (WMD of −2.69 (times/minute), 95% CI from −7.25 to 1.88; *p* = 0.25; I^2^ = 0%, Figure S5). Regarding the results of subgroup analyses, no significant differences were found in the comparisons of the heart rate during the heal lance with acupressure vs. usual care (WMD of −3.03 (times/minute), 95% CI from −9.80 to 3.75; *p* = 0.38; I^2^ = 0%), magnetic acupuncture vs. usual care (WMD of 9.10 (times/minute), 95% CI from −9.92 to 28.12; *p* = 0.35), or acupressure vs. nonacupoint stimulation intervention (WMD of −3.76 (times/minute), 95% CI from −10.28 to 2.76; *p* = 0.26; I^2^ = 41%).

#### 3.3.5. Heart Rate after Heel Lance

Five RCTs [24,25,26,28,29] evaluated the infants’ heart rate after heel lance; we pooled the findings using the fixed effects model and found that APS significantly improved heart rate after heel lance (WMD of −3.71 (times/minute), 95% CI from −7.23 to −0.18; *p* = 0.04; I^2^ = 0%). Subgroup analyses revealed that there were no differences in comparisons between acupuncture vs. usual care (WMD of −2.90 (times/minute), 95% CI from −28.46 to 22.66; *p* = 0.82), acupressure vs. usual care (WMD of −2.37 (times/minute), 95% CI from −9.07 to 4.34; *p* = 0.49; I^2^ = 0%), magnetic acupuncture vs. usual care (WMD of −0.40 (times/minute), 95% CI from −19.48 to 18.68; *p* = 0.97), acupressure vs. nonacupoint stimulation intervention (WMD of −3.89 (times/minute), 95% CI from −9.79 to 2.00; *p* = 0.20; I^2^ = 62%), or laser acupuncture vs. nonacupoint stimulation intervention (WMD of −5.08 (times/minute), 95% CI from −11.37 to 1.21; *p* = 0.11, Figure S6).

#### 3.3.6. Respiration Rate during Heel Lance

One RCT [29] reported the respiration rate during the heel lance procedure, and a significant difference was found in the APS cohort after pooling using the fixed effects model (WMD of −9.90 (times/minute), 95% CI from −11.93 to −7.88; *p* < 0.00001; I^2^ = 45%, Figure S7). The results of subgroup analyses revealed that the comparisons between acupressure vs. usual care (WMD of −11.38 (times/minute), 95% CI from −14.33 to −8.43; *p* < 0.00001) and acupressure vs. nonacupoint stimulation intervention (WMD of −9.90 (times/minute), 95% CI from −11.93 to −7.88; *p* < 0.00001) achieved statistical significances. 

#### 3.3.7. Respiration Rate after Heel Lance

Two RCTs [26,29] reported the respiration rate after heel lance, and no significant decrease in the respiration rate after heel lance was found in the APS cohort (fixed effects model, WMD of 1.91, 95% CI from −0.21 to 4.03; *p* = 0.08; I^2^ = 62%, Figure S8). In the subgroup analyses, the comparison between acupuncture vs. usual care revealed that the usual care cohort experienced a smaller increase in respiration rate than the acupuncture cohort. The differences between the respiration rates of acupressure vs. usual care (WMD of 0.06, 95% CI from −3.07 to 3.19; *p* = 0.97) and acupressure vs. usual care nonacupoint stimulation intervention (WMD of 2.00, 95% CI from −1.35 to 5.35; *p* = 0.24) were not significant. 

### 3.4. Tertiary Outcomes

#### Duration of Procedure

Five studies [22,25,27,28,29] used the duration of the procedure as a measurement of the effect of APS, and the pooling results did not show a significant improvement in the duration of the procedure in the APS groups compared to the control groups (random effects model, WMD of −15.94 (seconds), 95% CI from −35.14 to 3.25; *p* = 0.10; I^2^ = 86%). From the results of the subgroup analyses, significant differences were observed in the comparisons of acupressure vs. usual care (WMD of −29.31 (seconds), 95% CI from −57.32 to 1.31; *p* = 0.04; I^2^ = 69%) and laser acupressure vs. nonacupoint stimulation intervention (WMD of −18.43 (seconds), 95% CI from −31.31 to 5.55; *p* = 0.005). However, there were no significant differences in comparisons of the acupressure vs. nonacupoint stimulation intervention (WMD of 0.55 (seconds), 95% CI from −47.02 to 48.12; *p* = 0.98; I^2^ = 87%) or the NESAP vs. nonacupoint stimulation intervention (random effects model, WMD of −11.55 (seconds), 95% CI from −41.76 to 18.66; *p* = 0.45, Figure S9).

### 3.5. Meta-Regression Analyses and Publication Bias

Two outcomes—oxygenation concentration during heel lance and heart rate after heel lance—were subjected to further analyses by meta-regression (model: random effects; method: Sidik–Jonkman) because the pooling results were significant with low heterogeneity [41]. The meta-analysis found no significant relationships between outcomes and variables, such as neonate maturity (to term or not), APS interventions, practitioners, locations of acupoints, numbers of acupoints, meridians of acupoints, or between outcomes and the bias of the included studies, including allocation concealment, blinding of outcome assessment, blinding of participants and personnel, incomplete outcome data, selective reporting, and random sequence generation (Tables S1 and S2).

Symmetric funnel plot appearances were found for all measures. The Egger’s test *p* values of pain score during heel lance, oxygenation concentration during heel lance, oxygenation concentration after heel lance, heart rate during heel lance, heart rate after heel lance, respiratory rate during heel lance, and respiratory rate after heel lance were 0.3050, 0.9951, 0.5933, 0.5921, 0.5745, 0.4981, 0.0533, and 0.4404, respectively, and publication bias was noted. The trim and fill method was used to measure crying time and pain score after heel lance because publication bias could not be detected by Egger’s test, and the results of these outcomes were not changed after missing studies were added.

## 4. Discussion

Our results showed that APS could help improve some pain measures when the neonate accepts the heel lance, such as heart rate after the heel lance and respiratory rate during the heel lance. Five types of APS were used in our included studies, including acupuncture, acupressure, magnetic acupuncture, laser acupuncture, and NESAP. In the subgroups analyses, when the control group experienced usual care, acupuncture could alleviate the pain score during the heel lance procedure and decrease the crying time. Acupressure resulted in a decreased the pain score during and after the heel lance procedure, less oxygenation desaturation during the heel lance procedure, and a shorter procedure time of the heel lance when the control group experienced usual care. Laser acupuncture also meant that the heel lance procedure took less time when the control group experienced nonacupoint stimulation intervention. The effect of pain control from APS could be detected by reliability and validity measure tools when acupuncture and acupressure were used in neonates. The effect of pain relief from other types of APS can be seen in the changes in vital signs (oxygenation concentration, heart rate, and respiratory rate) and indirect parameters (procedure time). However, the use of acupuncture in the neonate may increase the infant’s respiratory rate after the heel lance procedure.

The effects of pain relief from APS have been observed not only by using evaluation tools but also in human brain images [6,42,43,44]. From the results of our meta-regression, the APS approaches did not affect the pooled results. The prescription of APS as a treatment is an important topic, but few studies have discussed it [45,46]. Because the use of acupoints has varied over hundreds of years, most RCTs use acupoints that have been demonstrated to be effective in previous studies, but they may add other acupoints [23]. Such study design means that it is difficult to identify effective prescriptions. Thus, we performed meta-regression using the theory of TCM to review the difference between the results and the prescriptions. Another advantage of this analysis is that we could use the fewest acupoints to achieve similar results. A concise prescription of APS is helpful for the practitioner.

Evidence of effective acupoints for treating neonatal pain is lacking. The studies we included utilized the following references to choose acupoints: no reference [21,22,23,26,30], books [27,28,30], and previous studies on pain control in neonates [24,28]. We used a textbook on acupuncture from the Chinese medical physician training program in Taiwan and published studies to examine the suitability of these acupoints. The textbook is named the *Color Book of Acupuncture and Moxibustion* [47] and includes several ancient traditional acupuncture books and clinical experiences edited following the standard acupoints declared by the World Health Organization. In the textbook, we noted that EX-HN3 could be used when children are agitated [26,27]; concurrent use of BL60 and KI3 [22,28] would be helpful to control heel pain; concurrent use of ST36 and KI3 [30] has not been reported, but they could treat leg and heel pain, respectively; LI4 [25] could be used in pain relief, but it is used to control pain in the head and neck; concurrent use of BL60, ST36, KI3, and SP6 [23] has not been noted; and the use of SP6 for pain improvement is mainly suggested for women. In conclusion, individual use of BL60, KI3, and ST36 could relieve heel or leg pain, and the concurrent use of BL60 and KI3 has also had similar effects, but the difference between concurrent and individual use was not significant according to the results of our meta-regression. However, a published RCT used BL60 and KI3 to achieve analgesic effects [28]. The use of battlefield acupuncture in neonates or pain control needs more evidence [24].

Meta-analyses have shown that positive effects can result from both expert practitioners and nonexperts [48] employing acupressure and auricular acupressure practices for pain relief [49]. However, evidence of therapeutic differences in APS could not be found. In clinical practice, these interventions must be practiced by doctors, which may cause the evidence to be lacking. Our results demonstrated that when neonates received APS, there was no difference in the results whether the practitioner was an expert or not. Although the pain score recorded for venipuncture was lower than that for heel lance, it is recommended that an expert practice these procedures [4]. This indicates that the APS procedure is easy to learn and practice after adequate training. In other words, when the analgesic effect is needed in a scenario where a medical agent or specialist in APS is unavailable, the APS intervention could still be performed and be effective. However, a clinical doctor or staff certified by the government may ensure the accuracy of APS and the safety of participants.

The definite mechanism of APS is still being explored, and there are two major mechanisms that could explain the analgesic effect: peripheral and central pain control theory [50]. There were several types of APS included in our analysis: acupuncture, acupressure, laser acupuncture, noninvasive electrical stimulation at acupuncture points (transcutaneous electrical nerve stimulation, TENS), and auricular acupressure [51]. Among them, acupuncture has been shown to have the ability to control pain through both peripheral and central pain control mechanisms, such as moderation of the pain threshold, anti-inflammation, endorphin elevation, less serotonin breakdown, and periaqueductal gray matter (PAGM) regulation [50]. Laser acupuncture has also been reported to relieve pain by decreasing inflammation and elevating endorphins [52]. Anti-inflammatory effects, increasing the pain threshold of pain, and endorphin and serotonin production are the main analgesic mechanisms of acupressure and auricular acupressure [51]. The increase in endorphins has been noted in TENS practice to improve pain [51]. Figure S10 shows the possible mechanisms of each APS intervention included in our analysis [50,51,52].

Although the methods of performing APS were different across our included studies, stimulation acupoint is the core procedure used for neonates. Most studies demonstrated their main APS technique but lacked a detailed protocol [21,22,23,25,26,28,29,30]. Ideas regarding the treatment dose of APS are still developing [53], but it is important for neonates and their caregivers because the information would be useful for forming an effective intervention. The duration of intervention, the time between practicing the intervention and the heel lance, and the content of the intervention are the three essential parts needed to present a comprehensive description of APS [53]. Furthermore, the use of acupoints is another important topic when APS is performed. Thus, it is recommended that the reference for selecting acupoints and the use of alternative techniques be disclosed. The above suggestions are also to be noted regarding the current RCT focus on the adult population [54]. Research using integrative interventions in neonates is just beginning. A clear and detailed description of APS protocol would help to elevate the quality of research.

Three kinds of staff practiced APS in our included studies: physician [22,26,27], study nurse [23], and trained researchers [24,28,29,30]. Most of the studies used non-invasive APS [21,22,23,24,25,28,29,30], for which the risk of complicated complications is low. However, the potential risks of skin infection (acupuncture) [46], skin irritation (acupuncture and laser acupuncture), and swallowing the magnetic balls (magnetic acupuncture) still exist when acupressure interventions are practiced [24]. Thus, a well-educated and experienced APS practitioner is the most recommended candidate for practicing APS. The absolute body surface area of the neonate is relatively small, which is an obstacle to locating the accurate acupoints. Furthermore, anatomical knowledge is also important for locating acupoints and avoiding dangerous regions in the human body. Only clinical, medical caregivers would have been taught this information during the training program. Although of the results of our meta-regression did not show a significant therapeutic difference between different APS practitioners, a Chinese medical physician or nurse who has received Chinese medical education provided by the government would confirm the quality of APS performance and the safety of neonates.

The types of APS practice were inconsistent, and we used meta-regression to examine the effect of different APS techniques on neonates who must undergo the heel lance procedure. The small number of included studies may have caused a limited ability to identify publication bias. Because heterogeneity may exist in the control group interventions, subgroup analyses were performed. Bias in the scoring of study quality and extractor bias can occur when the meta-analysis is performed. Thus, we used two authors to process the data extraction and quality assessment independently, and they followed the same rules in their work, which may have minimized the probability of bias attribution. Although our study has limitations, the results offer new findings on the effects of APS, along with important viewpoints that can benefit future RCT design.

## 5. Conclusions

As far as we know, this is the first study to evaluate the effect of APS on controlling heel lance pain in neonates through meta-analysis. According to our results, APS could improve infant’s oxygenation concentration during heel lance, heart rate after heel lance, and respiratory rate during heel lance. The variables of neonate maturity, APS intervention, practitioner, locations of acupoints, number of acupoints, meridians of acupoints, and bias summary did not affect the results regarding oxygenation concentration during heel lance or heart rate after heel lance. Compared to the usual care cohort, the acupuncture and acupressure cohorts demonstrated significant effects on pain score, oxygenation saturation during heel lance procedure, and procedure time, and on pain score during and after the procedure, oxygenation saturation during the procedure, and procedure time, respectively. A shorter procedure time was also found when the neonate received lase acupuncture, compared to the nonacupoint stimulation cohort. APS prescription and detailed protocol demonstrations are needed to design further trials to comprehensively study the APS mechanisms in neonates.

## Figures and Tables

**Table 1:** The characteristics of RCTs.

RCTs (Country)	Neonates	Numbers (Pre-/Post-)	Study Arms (Prescription)	Control	Acupoints (Position)	Outcome Measurements
			Practitioner			
Gottschling et al., 2010 (Germany) [21]	Term	IC: 25/25 CC: 25/25	Laser acupuncture (dose: 0.45 J; duration: not mentioned; interval: not mentioned)	Placebo laser acupuncture	LI4 (hand), Shenmen (ear)	Pain score: PIPP (during procedure)
			Not mentioned			
Ecevit et al., 2011 (Turkey) [26]	Preterm	IC: 10/10 CC1: 30/30	Acupuncture (duration: 30 min; interval: not mentioned)	Usual care	EX-HN3 (head)	Pain score: NIPS (during procedure) Crying time Oxygen saturation (after procedure) HR (after procedure) RR (after procedure)
			Competent doctor			
Abbasoğlu et al., 2015a (Turkey) [22]	Preterm	IC: 16/16 CC: 16/16	Acupressure (duration: 3 min; interval: immediately)	Usual care	BL60 (leg), KI3 (leg)	Pain score: PIPP (during procedure) Crying time Procedure time
			Trained physician			
Abbasoğlu et al., 2015b (Turkey) [27]	Term	IC: 21/21 CC: 21/21	Laser acupuncture (dose: 0.3 J; duration: 0.5 min; interval: 2 min)	Sucrose (dose: 0.5 mL of 24% sucrose; interval: 2 min)	EX-HN3 (head)	Pain score: PIPP (during procedure) Crying time Duration of procedure
			Trained physician			
Mitchell et al., 2016 (USA) [23]	Term	IC1: 42/37 IC2: 41/40 CC1: 39/37 CC2: 40/39	IC1: NESAP (dose: 3.5 mA, 10 Hz; duration: 10 ± 1 min; interval: uninterrupted) IC2: NESAP + sucrose (dose: 1 ± 0.1 mL of the 24% sucrose; interval: 2 min)	CC1: sucrose (dose: 1.0 mL of 24% sucrose; interval: 2 min) CC2: usual care	ST36 (leg), SP6 (leg), BL60 (leg), KI3 (leg)	Pain score: PIPP (during/after procedure)
			Research nurse			
Chen et al., 2017 (Australia) [24]	Preterm+ term	IC: 21 CC: 19	Magnetic acupuncture (dose: 100 G, 1.7 mm; duration: 3 days; interval: 2 h)	Usual care	Cingulate Gyrus, Thalamus, Omega, Point Zero, Shenmen (ear)	Pain score: PIPP (during/after procedure) HR (during/after procedure) Oxygenation saturation (during/after procedure)
			Experienced practitioner			
Özkan et al., 2019 (Turkey) [28]	Term	IC: 47/47 CC1: 47/47 CC2: 46/46	Acupressure (duration: 2 min; interval: 5 min)	CC1: massage (duration: 2 min; interval: 5 min) CC2: usual care	BL60 (leg), KI3 (leg)	Pain score: NIPS (during/after procedure) Oxygenation saturation (during/after procedure) HR (during/after procedure) Crying time Duration of procedure
			Certificated researchers			
Deniz et al., 2021 (Turkey) [30]	Term	IC: 35/35 CC1: 35/35 CC2: 35/35	Acupressure (duration: 7 min; interval: not mentioned)	CC1: reflexology (duration: 7 min; interval: not mentioned) CC2: usual care	ST36 (leg), KI3 (leg)	Pain score: N-PASS (during procedure) Crying time
			Certificated researchers			
Stadler et al., 2021 (Austria) [25]	Term	IC: 48/44 CC: 48/47	Laser acupuncture (dose: 0.6 J; duration: 2 min; interval: 2.5 min)	Glucose (mL of the 30% glucose not mentioned)	LI4 (hand)	Pain score: PIPP (during procedure) HR (after procedure) Oxygenation saturation (after procedure) Crying time Duration of procedure
			Not mentioned			
Ibrahim et al., 2022 (Egypt) [29]	Term	IC: 40/40 CC1: 40/40 CC2: 40/40	Acupressure (duration: 2 min; interval: not mentioned)	CC1: massage (duration: 2 min; interval: not mentioned) CC2: usual care	Not mentioned	Pain score: NIPS (during/after procedure) HR (during/after procedure) RR (during/after procedure) Oxygenation saturation (during/after procedure) Duration of procedure
			Researchers			

RCT: randomized control trials; IC: intervention cohort; CC: control cohort; J: joule; LI: large intestine meridian; PIPP: Premature Infant Pain Profile; EX-HN: extra points of head and neck; NIPS: Neonatal Infant Pain Scale; HR: heart rate; RR: respiration rate; BL: bladder meridian; KI: kidney meridian; NESAP: noninvasive electrical stimulation at acupuncture points; mA: milliampere; Hz: Hertz; mL: milliliter; ST: stomach meridian; SP: spleen meridian; N-PASS: Neonatal Pain, Agitation, and Sedation Scale; G: Gaussian; mm: millimeter.

## Data Availability

The data from this study are available from the corresponding author upon reasonable request.

## Supplementary Materials

The following supporting information can be downloaded at: https://www.mdpi.com/article/10.3390/children10061024/s1, Figure S1: Preferred Reporting Items for Systematic Reviews and Meta-Analyses (PRISMA) statement of search results; Figure S2: Risk of bias graph and risk of bias summary; Figure S3: Forest plot of oxygenation concentration during heel lance procedure; Figure S4: Forest plot of oxygenation concentration after procedure; Figure S5: Forest plot of heart rate during heel lance procedure; Figure S6: Forest plot of heart rate after heel lance procedure; Figure S7: Forest plot of respiratory rate during heel lance procedure; Figure S8: Forest plot of respiratory rate after heel lance procedure; Figure S9: Forest plot of duration of heel lance; Figure S10: The possible mechanism of acupoint stimulation in pain control; Table S1: Report of univariate moderator analyses in oxygenation concentration (during procedure); Table S2: Report of univariate moderator analyses in heart rate (after procedure).
