# Supplementary material for: Effect of Acupoint Stimulation on Controlling Pain from Heel Lance in Neonates: A Systematic Review and Meta-Analysis of Randomized Controlled Trials
Source: Children (Basel). 2023 Jun 7;10(6):1024. doi: 10.3390/children10061024 (PMC10297571; doi:10.3390/children10061024)
Supplement: Supplementary file 1 [file children-10-01024-s001.zip › children-2343118-supplementary.pdf]

Table S1. Report of univariate moderator analyses in oxygenation concentration (during procedure).

Moderator	Coefficient	SE	95% CI	Z	*p*-value
Term (Yes=1, other=0)	0.061	0.468	-0.857 to -0.978	0.13	0.896
Acupressure (Yes=1, other=0)	0.061	0.468	-0.857 to -0.978	0.13	0.896
Trained practitioner (Yes=1, other=0)	0.155	0.357	-0.545 to 0.855	0.43	0.664
Acupoints at head and neck (Yes=1, other=0)	-0.061	0.468	-0.978 to 0.857	-0.13	0.896
Acupoints at leg (Yes=1, other=0)	0.188	0.351	-0.499 to 0.875	0.54	0.592
Numbers of acupoints (≥2=1, other=0)	0.155	0.357	-0.545 to 0.855	0.43	0.664
Numbers of acupoints (≥5=1, other=0)	-0.061	0.468	-0.978 to 0.857	-0.13	0.896
Acupoints at BL (Yes=1, other=0)	0.188	0.351	-0.499 to 0.875	0.54	0.592
Acupoints at KI (Yes=1, other=0)	0.188	0.351	-0.499 to 0.875	0.54	0.592
Acupoints at BL+KI (Yes=1, other=0)	0.188	0.351	-0.499 to 0.875	0.54	0.592
Random sequence generation (Low risk=1, other=0)	0.155	0.357	-0.545 to 0.855	0.43	0.664
Allocation concealment (Low risk=1, other=0)	0.155	0.357	-0.545 to 0.855	0.43	0.664
Blinding of outcome assessment (Low risk=1, other=0)	-0.061	0.468	-0.978 to 0.857	-0.13	0.896

BL: bladder meridian; KI: kidney meridian

Table S2. Report of univariate moderator analyses in heart rate (after procedure).

Moderator	Coefficient	SE	95% CI	Z	*p*-value
Term (Yes=1, other=0)	-0.183	0.366	-0.901 to -0.535	-0.50	0.617
Acupuncture (Yes=1, other=0)	0.123	0.511	-0.879 to 1.126	0.24	0.809
Laser acupuncture (Yes=1, other=0)	-0.139	0.351	-0.827 to 0.548	-0.40	0.691
Acupressure (Yes=1, other=0)	-0.050	0.283	-0.606 to 0.505	-0.18	0.859
Trained *practitioner* (Yes=1, other=0)	-0.026	0.277	-0.570 to 0.517	-0.10	0.924
Acupoints at head and neck (Yes=1, other=0)	0.206	0.339	-0.458 to 0.870	0.61	0.542
Acupoints at leg (Yes=1, other=0)	-0.185	0.292	-0.758 to 0.389	-0.63	0.527
Numbers of acupoints (≥2=1, other=0)	-0.063	0.279	-0.610 to 0.483	-0.23	0.820
Numbers of acupoints (≥5=1, other=0)	0.224	0.414	-0.588 to 1.036	0.54	0.589
Acupoints at BL (Yes=1, other=0)	-0.185	0.292	-0.758 to 0.389	-0.63	0.527
Acupoints at KI (Yes=1, other=0)	-0.185	0.292	-0.758 to 0.389	-0.63	0.527
Acupoints at LI (Yes=1, other=0)	-0.139	0.351	-0.827 to 0.548	-0.40	0.691
Acupoints at BL+KI (Yes=1, other=0)	-0.185	0.292	-0.758 to 0.389	-0.63	0.527
Random sequence generation (Low risk=1, other=0)	-0.159	0.283	-0.713 to 0.395	-0.56	0.574
Allocation concealment (Low risk=1, other=0)	-0.159	0.283	-0.713 to 0.395	-0.56	0.574
Blinding of outcome assessment (Low risk=1, other=0)	0.014	0.299	-0.572 to 0.600	0.05	0.963

BL: bladder meridian; KI: kidney meridian

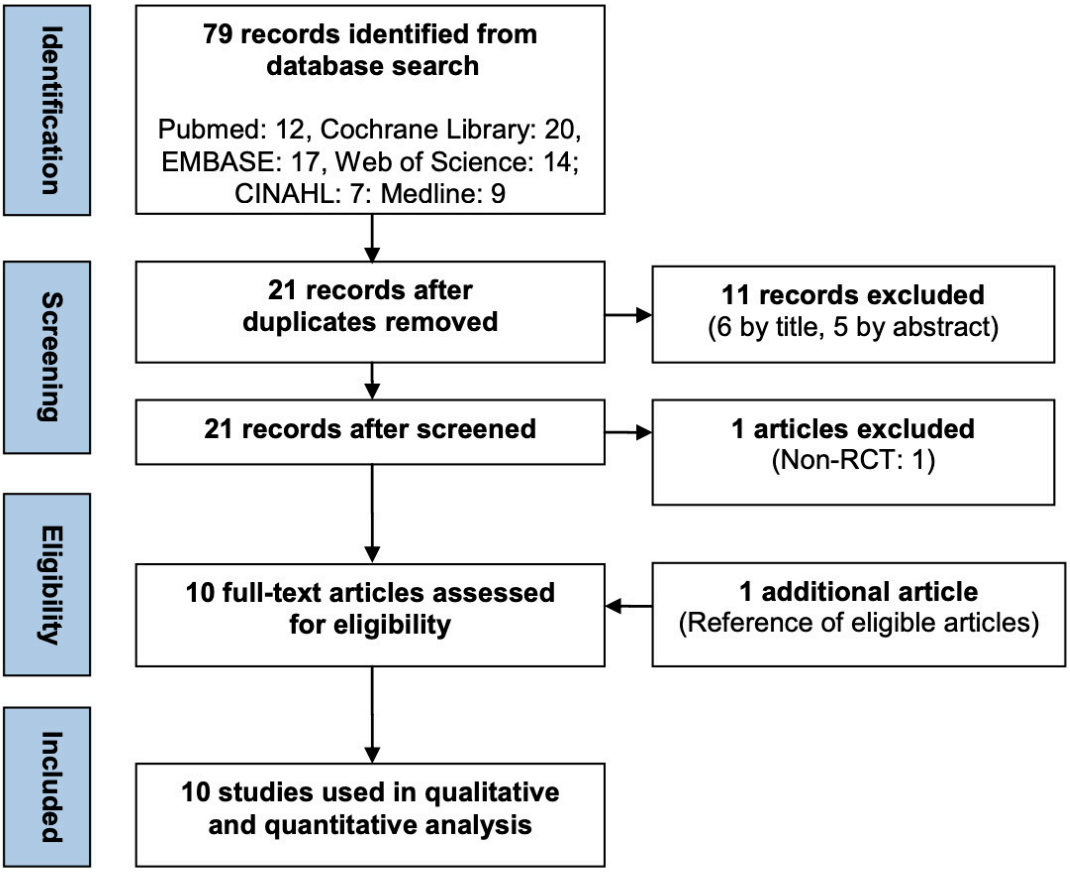

Figure S1. Preferred Reporting Items for Systematic Reviews and Meta-Analyses (PRISMA) statement of search results.

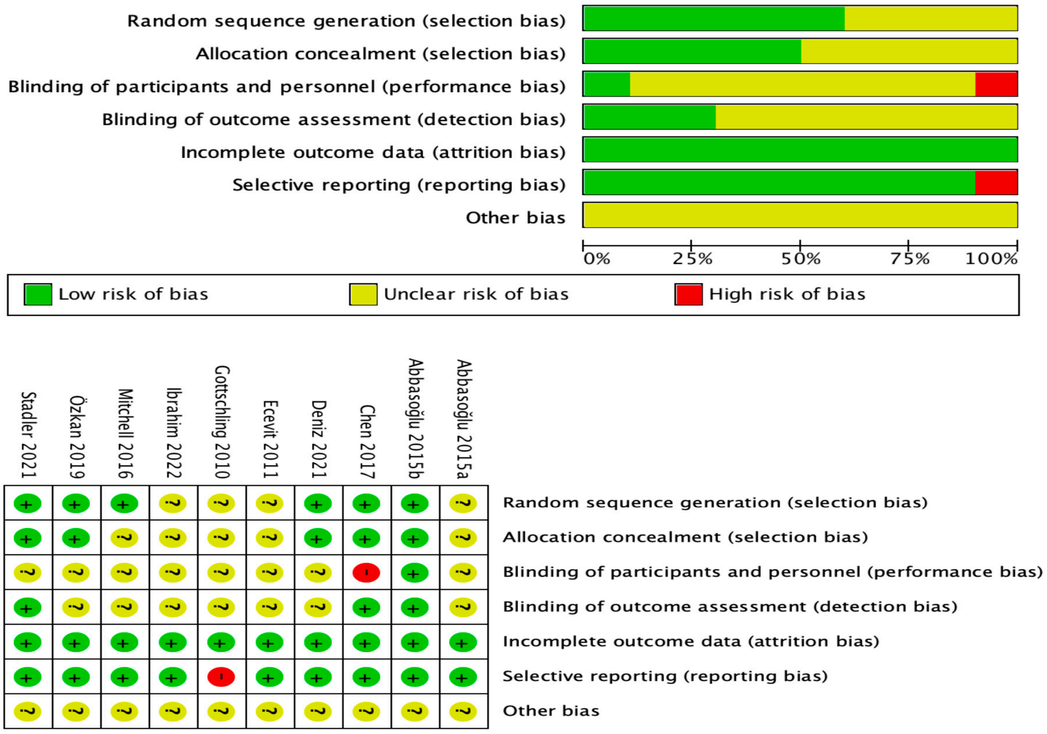

Figure S2. Risk of bias graph and risk of bias summary.

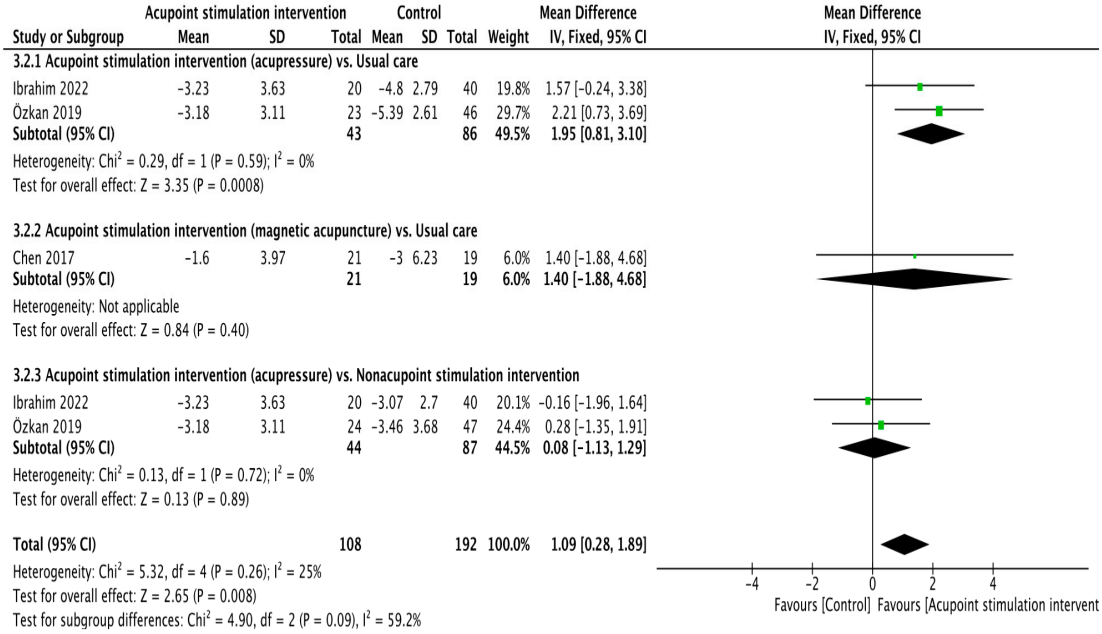

Figure S3. Forest plot of oxygenation concentration during heel lance procedure.

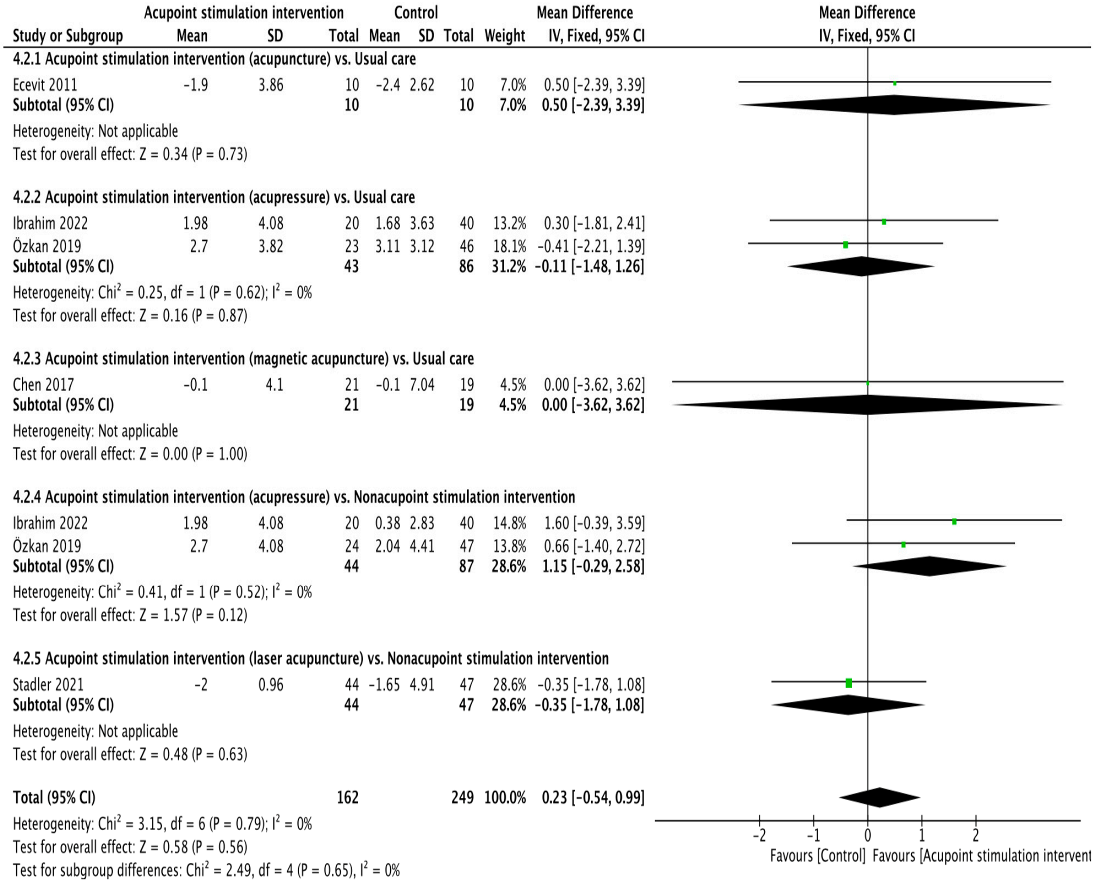

Figure S4. Forest plot of oxygenation concentration after procedure.

Study or Subgroup	Acupoint stimulation intervention Mean	SD	Total	Control Mean	SD	Total	Weight	Mean Difference IV, Fixed, 95% CI
**5.2.1 Acupoint stimulation intervention (acupressure) vs. Usual care**								
Ibrahim 2022	17.62	20.58	20	22.52	14.3	40	20.6%	−4.90 [−14.95, 5.15]
Özkan 2019	26.39	18.41	23	27.85	18.22	46	24.7%	−1.46 [−10.64, 7.72]
**Subtotal (95% CI)**			**43**			**86**	**45.3%**	**−3.03 [−9.80, 3.75]**
Heterogeneity: $Chi^2 = 0.25$, df = 1 (P = 0.62); $I^2 = 0\%$								
Test for overall effect: Z = 0.87 (P = 0.38)								
**5.2.2 Acupoint stimulation intervention (magnetic acupuncture) vs. Usual care**								
Chen 2017	26.1	24.49	21	17	35.32	19	5.8%	9.10 [−9.92, 28.12]
**Subtotal (95% CI)**			**21**			**19**	**5.8%**	**9.10 [−9.92, 28.12]**
Heterogeneity: Not applicable								
Test for overall effect: Z = 0.94 (P = 0.35)								
**5.2.3 Acupoint stimulation intervention (acupressure) vs. Nonacupoint stimulation intervention**								
Ibrahim 2022	17.62	20.58	20	26.52	14.95	40	20.2%	−8.90 [−19.04, 1.24]
Özkan 2019	26.39	18.41	24	26.52	14.95	47	28.7%	−0.13 [−8.65, 8.39]
**Subtotal (95% CI)**			**44**			**87**	**49.0%**	**−3.76 [−10.28, 2.76]**
Heterogeneity: $Chi^2 = 1.69$, df = 1 (P = 0.19); $I^2 = 41\%$								
Test for overall effect: Z = 1.13 (P = 0.26)								
**Total (95% CI)**			**108**			**192**	**100.0%**	**−2.69 [−7.25, 1.88]**
Heterogeneity: $Chi^2 = 3.52$, df = 4 (P = 0.48); $I^2 = 0\%$								
Test for overall effect: Z = 1.15 (P = 0.25)								
Test for subgroup differences: $Chi^2 = 1.59$, df = 2 (P = 0.45), $I^2 = 0\%$								

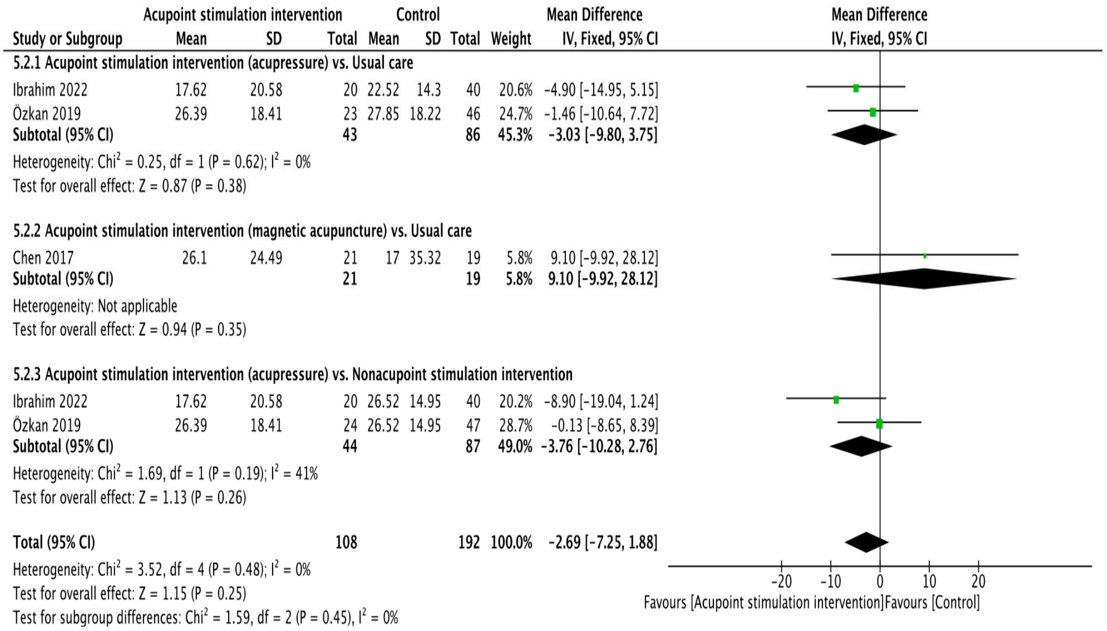

Figure S5. Forest plot of heart rate during heel lance procedure.

Study or Subgroup	Acupoint stimulation intervention Mean	SD	Total	Control Mean	SD	Total	Weight	Mean Difference IV, Fixed, 95% CI
**7.2.1 Acupoint stimulation intervention (acupuncture) vs. Usual care**								
Ecevit 2011	13.7	33.93	10	16.6	23.45	10	1.9%	−2.90 [−28.46, 22.66]
**Subtotal (95% CI)**			**10**			**10**	**1.9%**	**−2.90 [−28.46, 22.66]**
Heterogeneity: Not applicable								
Test for overall effect: Z = 0.22 (P = 0.82)								
**7.2.2 Acupoint stimulation intervention (acupressure) vs. Usual care**								
Ibrahim 2022	−8.83	21.25	20	−3.5	6.1	40	13.7%	−5.33 [−14.83, 4.17]
Özkan 2019	−17.15	18.87	23	−17.72	18.96	46	13.9%	0.57 [−8.89, 10.03]
**Subtotal (95% CI)**			**43**			**86**	**27.6%**	**−2.37 [−9.07, 4.34]**
Heterogeneity: $Chi^2 = 0.74$, df = 1 (P = 0.39); $I^2 = 0\%$								
Test for overall effect: Z = 0.69 (P = 0.49)								
**7.2.3 Acupoint stimulation intervention (magnetic acupuncture) vs. Usual care**								
Chen 2017	−2.5	25.28	21	−2.1	34.96	19	3.4%	−0.40 [−19.48, 18.68]
**Subtotal (95% CI)**			**21**			**19**	**3.4%**	**−0.40 [−19.48, 18.68]**
Heterogeneity: Not applicable								
Test for overall effect: Z = 0.04 (P = 0.97)								
**7.2.4 Acupoint stimulation intervention (acupressure) vs. Nonacupoint stimulation intervention**								
Ibrahim 2022	−8.83	21.25	20	−11.04	4.41	40	14.0%	2.21 [−7.20, 11.62]
Özkan 2019	−17.15	18.87	24	−9.32	1.51	47	21.7%	−7.83 [−15.39, −0.27]
**Subtotal (95% CI)**			**44**			**87**	**35.7%**	**−3.89 [−9.79, 2.00]**
Heterogeneity: $Chi^2 = 2.66$, df = 1 (P = 0.10); $I^2 = 62\%$								
Test for overall effect: Z = 1.29 (P = 0.20)								
**7.2.5 Acupoint stimulation intervention (laser acupuncture) vs. Nonacupoint stimulation intervention**								
Stadler 2021	−1.73	8.93	44	3.35	19.97	47	31.4%	−5.08 [−11.37, 1.21]
**Subtotal (95% CI)**			**44**			**47**	**31.4%**	**−5.08 [−11.37, 1.21]**
Heterogeneity: Not applicable								
Test for overall effect: Z = 1.58 (P = 0.11)								
**Total (95% CI)**			**162**			**249**	**100.0%**	**−3.71 [−7.23, −0.18]**
Heterogeneity: $Chi^2 = 3.86$, df = 6 (P = 0.70); $I^2 = 0\%$								
Test for overall effect: Z = 2.06 (P = 0.04)								
Test for subgroup differences: $Chi^2 = 0.46$, df = 4 (P = 0.98), $I^2 = 0\%$								

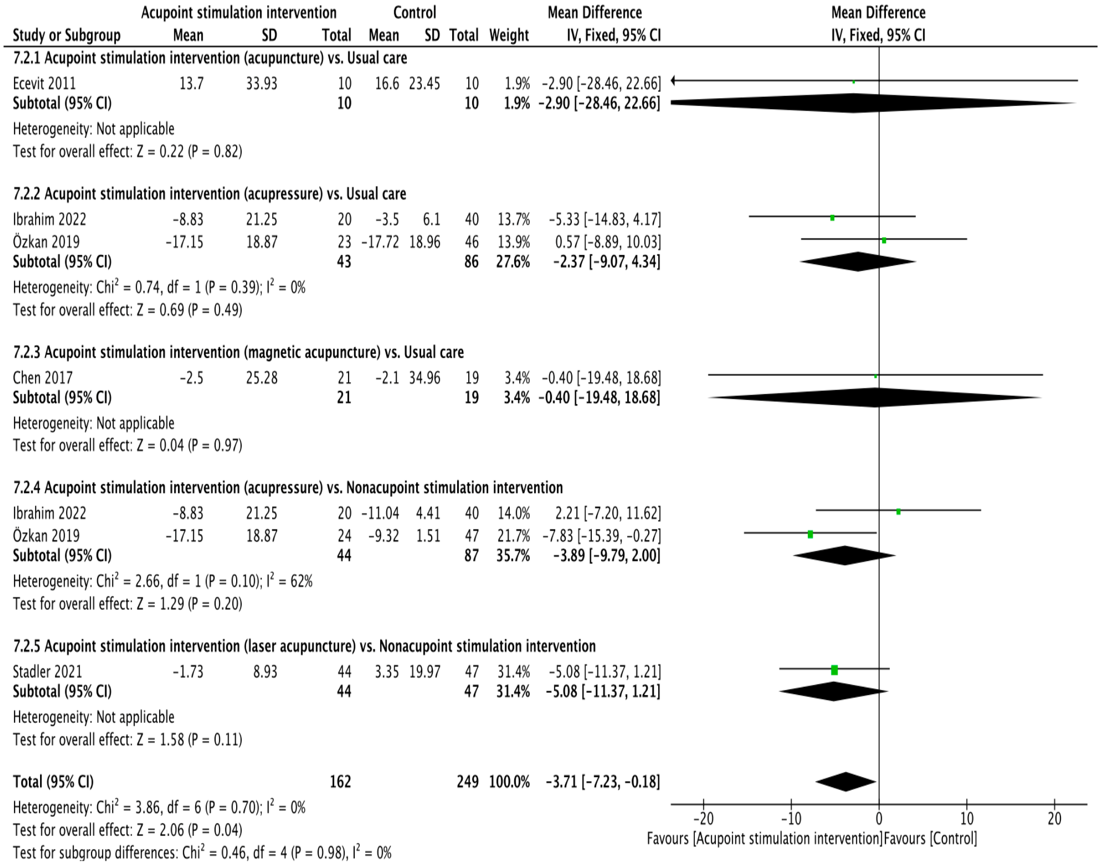

Figure S6. Forest plot of heart rate after heel lance procedure.

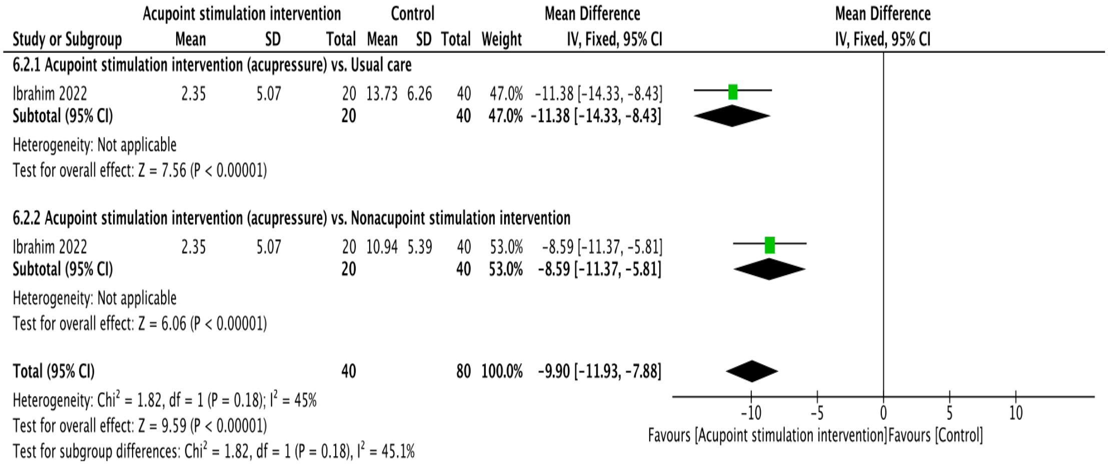

Figure S7. Forest plot of respiratory rate during heel lance procedure.

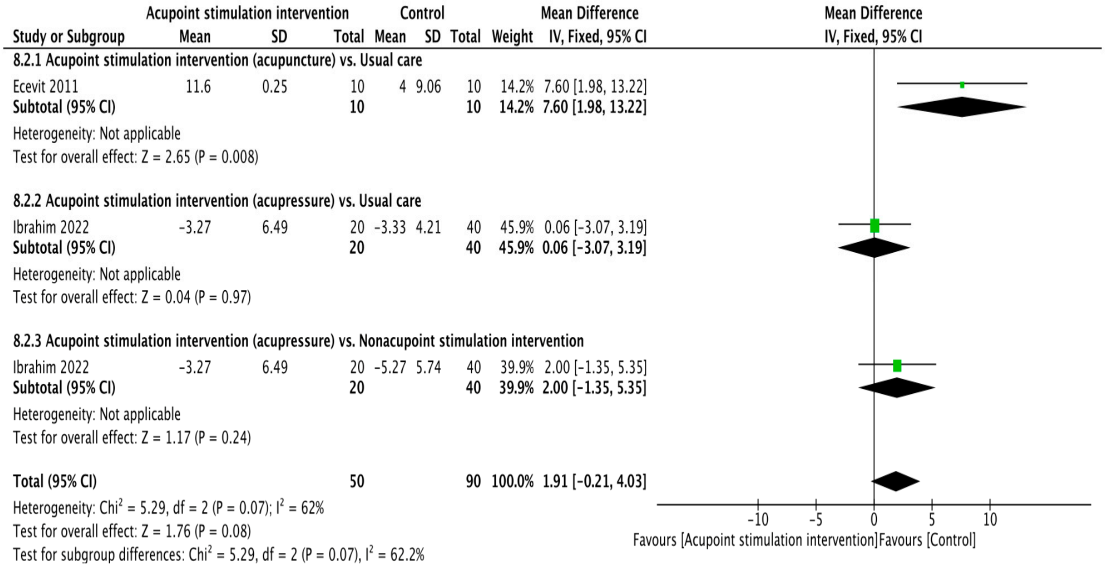

Figure S8. Forest plot of respiratory rate after heel lance procedure.

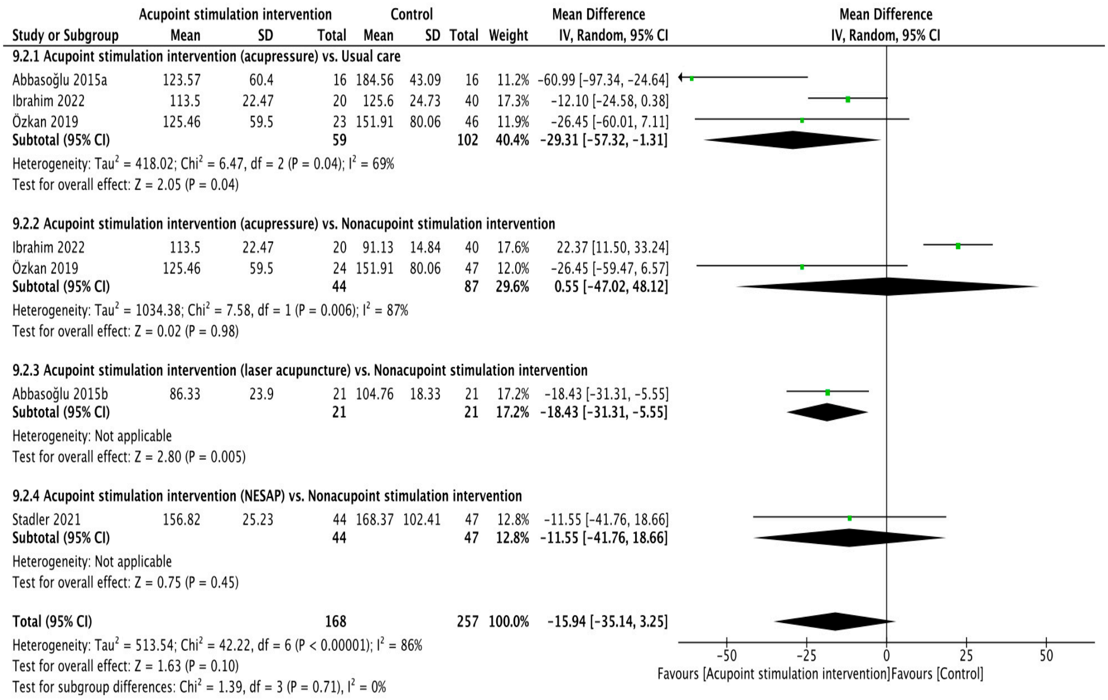

Figure S9. Forest plot of duration of heel lance.

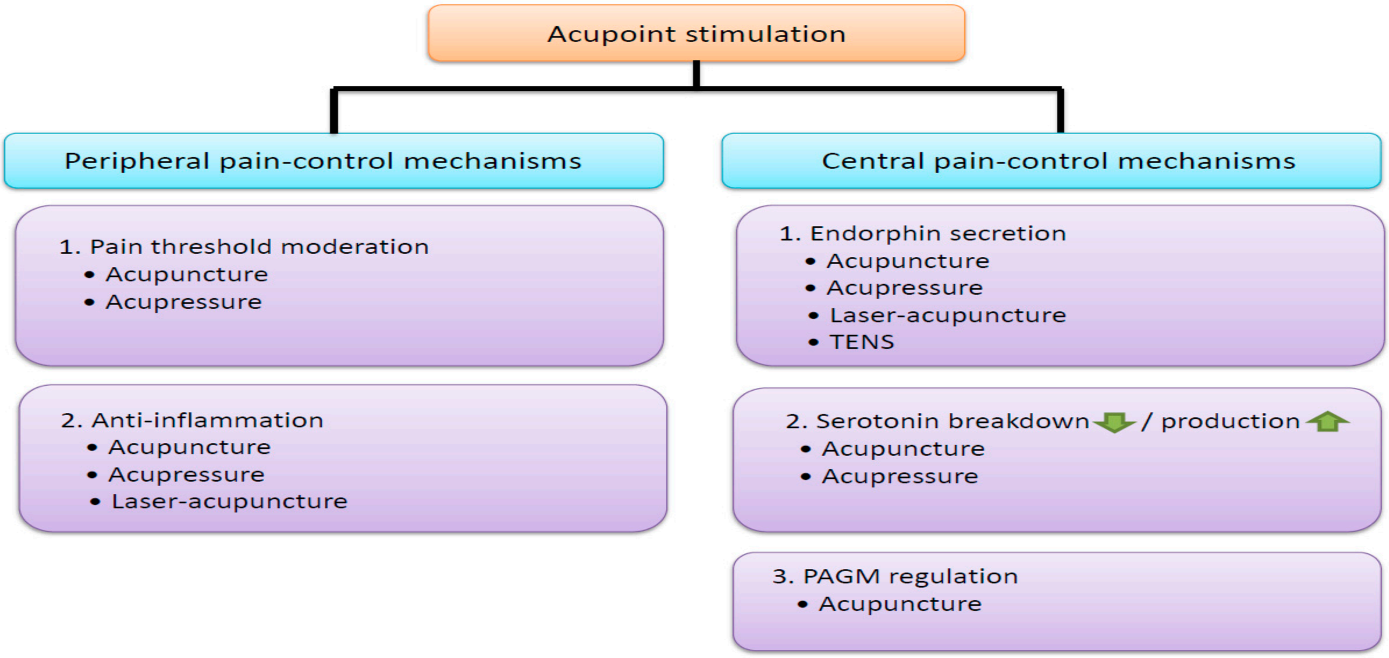

TENS: transcutaneous electrical nerve stimulation; PAGM: periaqueductal gray matter.

Figure S10. The possible mechanism of acupoint stimulation in pain control.
